# Targeted Perfusion Therapy in Spinal Cord Trauma

**DOI:** 10.1007/s13311-019-00820-6

**Published:** 2020-01-08

**Authors:** Samira Saadoun, Marios C. Papadopoulos

**Affiliations:** Academic Neurosurgery Unit, St. George’s University of London, Cranmer Terrace, Tooting, London, SW17 0RE UK

**Keywords:** Blood pressure, critical care, decompression, dura, monitoring, spinal cord injury

## Abstract

We review state-of-the-art monitoring techniques for acute, severe traumatic spinal cord injury (TSCI) to facilitate targeted perfusion of the injured cord rather than applying universal mean arterial pressure targets. Key concepts are discussed such as intraspinal pressure and spinal cord perfusion pressure (SCPP) at the injury site, respectively, analogous to intracranial pressure and cerebral perfusion pressure for traumatic brain injury. The concept of spinal cord autoregulation is introduced and quantified using spinal pressure reactivity index (sPRx), which is analogous to pressure reactivity index for traumatic brain injury. The U-shaped relationship between sPRx and SCPP defines the optimum SCPP as the SCPP that minimizes sPRx (i.e., maximizes autoregulation), and suggests that not only ischemia but also hyperemia at the injury site may be detrimental. The observation that optimum SCPP varies between patients and temporally in each patient supports individualized management. We discuss multimodality monitoring, which revealed strong correlations between SCPP and injury site metabolism (tissue glucose, lactate, pyruvate, glutamate, glycerol), monitored by surface microdialysis. Evidence is presented that the dura is a major, but unappreciated, cause of spinal cord compression after TSCI; we thus propose expansion duroplasty as a novel treatment. Monitoring spinal cord blood flow at the injury site has revealed novel phenomena, e.g., 3 distinct blood flow patterns, local steal, and diastolic ischemia. We conclude that monitoring from the injured spinal cord in the intensive care unit is a safe technique that appears to enable optimized and individualized spinal cord perfusion.

**Electronic supplementary material:**

The online version of this article (10.1007/s13311-019-00820-6) contains supplementary material, which is available to authorized users.

Each year, about 23 million people worldwide have a traumatic spinal cord injury (TSCI) [1], which is a major, life-changing event that leaves most people paralyzed or wheelchair bound. Apart from paralysis, TSCI also causes loss of sensation, loss of voluntary control of the urinary bladder and bowel, sexual dysfunction, and, in the case of cervical TSCI, impaired breathing and thermoregulation as well as hypotension. Other than the human suffering, there are economic implications; e.g., in the USA, the estimated lifetime cost of caring for a patient with severe cervical TSCI is estimated at $1,400,000, excluding loss of income [2]. Currently, there is no treatment proven to improve outcome after TSCI [3]. Here, we discuss novel concepts that may improve the clinical management of acute TSCI.

## Current Management

### Surgical

After TSCI, patients are transferred to neurosurgical or orthopedic units and most undergo spinal surgery to correct deformity and stabilize the fractured spine by placing screws, plates, and rods. Several surgical controversies exist, e.g., anterior *versus* posterior approach, number of levels to be fixed, timing of surgery, and the role of laminectomy [4–10]; thus, operative management largely relies on surgeons’ preferences rather than robust evidence. Substantial literature has been devoted to the timing and role of surgery; currently, most surgeons opt for early surgery, once the patient is medically stable [8, 10].

### Anesthetic and Medical

TSCI is a multisystem disease that impairs ventilation and causes pulmonary infections (from diaphragmatic or intercostal muscle paralysis), bradycardia and hypotension (from damage to sympathetic cord pathways), as well as decubitus ulcers and deep venous thrombosis (from immobility) [11]. Patients with cervical or upper thoracic TSCI are generally admitted in intensive care units (ICUs) where there is wide variability in their management. For example, in the UK, the optimum blood pressure to maintain is unclear [4], whereas in the USA, the AANS/CNS joint guidelines are followed. These guidelines recommend maintaining mean arterial pressure (MAP) at 85–90 mmHg for the first week after TSCI, but without robust evidence of benefit [12]. There is no consensus on the use of arterial or central venous lines during surgery and in ICU and in the type of vasopressor or anesthetic to be administered [4].

## Analogy with Traumatic Brain Injury

The management of traumatic brain injury (TBI) fundamentally differs from the management of TSCI. Patients with severe TBI are intubated and transferred to an ICU where probes are inserted intracranially to monitor parameters such as intracranial pressure (ICP), cerebral perfusion pressure (CPP = MAP minus ICP), vascular pressure reactivity index (PRx), optimum CPP (CPP_opt_), as well as injury site metabolites using microdialysis (MD), tissue oxygen, and spreading depolarizations [13, 14]. Though the extent of monitoring varies between ICUs, 2 parameters (ICP and CPP) are a key to the TBI management and are commonly monitored [14]. The focus is to reduce ICP and increase CPP to prevent secondary brain damage from cerebral ischemia and brain herniation. There are several treatments to reduce ICP and increase CPP, e.g., osmotic diuretics, reducing arterial pCO_2_, increasing the dose of vasopressors, cerebrospinal fluid (CSF) drainage, hypothermia, barbiturates, evacuation of hematoma, and decompressive craniectomy [14–16]. These treatments are widely used in TBI patients, but their efficacy in TSCI is largely unexplored. Until recently, the lack of spinal cord monitoring in TSCI patients has made it impossible to evaluate the effect of such therapies on spinal cord physiology and metabolism.

## Monitoring Spinal Cord Pressure

### Monitoring Technique

**Fig. 1:**
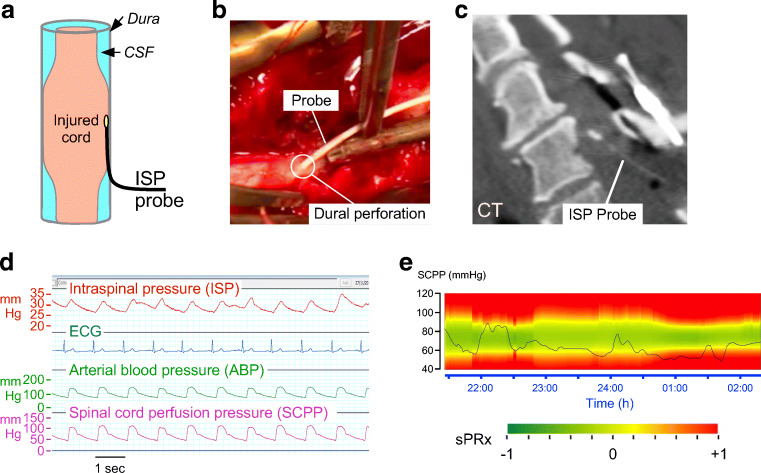
ISP monitoring. (**a**) Schematic showing ISP probe between swollen cord and dura. (**b**) Intraoperative photo taken as an ISP probe was inserted intradurally. (**c**) Postoperative CT. (**d**) Photo of computer screen showing monitored signals including ISP, ECG, ABP, and SCPP. SCPP is ABP minus ISP. (**e**) Enhanced visualization of SCPP *versus* time. SCPP (dark line). Range of optimal (green), intermediate (yellow), and suboptimal (red) SCPPs, with sPRx color scale.

In 2014, we described a technique (Fig. 1a–d) for placing a probe intradurally at the injury site to monitor the pressure of the injured cord as it is compressed against surrounding structures, which we termed intraspinal pressure (ISP) [17, 18]. We monitor ISP using the Codman ICP probe, because its cable is thinner and longer than that of other probes, e.g., Camino, and is licensed for use in humans. The probe is inserted intraoperatively during posterior surgical approach to the spine. Under the operating microscope, after laminotomy or laminectomy, the theca is perforated 1 spinal level below the injury with a 90°-angled needle to avoid damaging the underlying cord; the perforation is then widened with a blunt hook. The probe is tunneled into the wound, inserted through the thecal perforation, and advanced parallel to the cord to the point of maximal cord compression based on pressure measurements and the preoperative MRI. Several techniques reduce the risk of postoperative CSF leak and wound infection: The skin is sutured with nylon and sprayed with Opsite, a silk suture is used to tighten the skin around the probe, a wound drain is placed on gravity to divert CSF away from the wound, and an Ioban drape is adhered over the wound and probe exit site. Data from 42 patients indicate that ISP monitoring is safe [19]. The major drawback of ISP monitoring is that the probe can only be inserted during a posterior surgical approach. Depending on the location of the spine fracture and the surgeon’s preference, acute spinal decompression and fusion may be done from an anterior approach, which would preclude probe insertion.

### Physiological Parameters

The ISP waveform is similar to ICP with 3 characteristic peaks (P1 percussion, P2 tidal, P3 dicrotic) and comparable Fourier spectra that have prominent cardiac and respiratory peaks. By analogy with CPP for TBI, in the TSCI patients, we compute the spinal cord perfusion pressure (SCPP) as MAP-ISP (Table 1). SCPP more accurately measures cord perfusion than the currently used MAP because patients with the same MAP have different SCPPs depending on ISP. Aiming to optimize SCPP, rather than MAP, in TSCI is the first step towards individualized patient management.

**Table 1:** Comparison of physiological parameters in TSCI and TBI

TSCI	
ISP	Intraspinal pressure
SCPP	Spinal cord perfusion pressure
sPRx	Spinal pressure reactivity index
sRAP	Spinal compensatory reserve
SCPP_opt_	Optimum spinal cord perfusion pressure
TBI	
ICP	Intracranial pressure
CPP	Cerebral perfusion pressure
PRx	Pressure reactivity index
RAP	Compensatory reserve
CPP_opt_	Optimum cerebral perfusion pressure

TBI = traumatic brain injury; TSCI = traumatic spinal cord injury

### Optimum SCPP

We applied the concept of vascular pressure reactivity, based on the autoregulation curve for brain [20], to the injured cord to obtain the spinal pressure reactivity index (sPRx) as the running correlation coefficient between ISP and MAP [17, 18]. sPRx ranges from − 1 to + 1; sPRx ≤ 0 indicates intact vascular reactivity, whereas sPRx > 0 indicates impaired reactivity. sPRx plotted against SCPP, using data from several TSCI patients, yields a U-shaped curve, which defines the optimum SCPP (SCPP_opt_) as the SCPP that minimizes sPRx and is similar to the PRx *versus* CPP plot for TBI [17, 18, 21]. The concept of SCPP_opt_ is clinically important because it suggests that not only hypoperfusion but also hyperperfusion at the injury site may be detrimental. Potential mechanisms of hyperperfusion-induced cord injury include cord swelling, cord hemorrhage, and a local steal phenomenon [22].

sPRx *versus* SCPP plots for individual patients, rather than pooled patient data, yield SCPP_opt_ values that differ markedly, by up to 60 mmHg, between patients [23]. The patient-dependent SCPP_opt_ suggests that individualized patient management is required to achieve targeted perfusion therapy. This makes sense because several factors, which differ between TSCI patients, likely determine SCPP_opt_, e.g., level and mechanism of injury, extent of microvascular damage, tissue ischemia, tissue acidosis, and pre-TSCI baseline blood pressure. Two further modifications were introduced to refine the concept of SCPP_opt_: First, since many SCPP_opt_-determining factors vary with time (e.g., cord ischemia, edema, or acidosis), SCPP_opt_ must also vary with time in each patient. Second, there is often a range of SCPPs, rather than a single value (minimum of the sPRx *versus* SCPP curve), associated with intact autoregulation. We thus extended the concept of SCPP_opt_ to represent a range of pressures, computed from a sliding window of the preceding 4 h, updated each minute [23]. An enhanced display technique allows the SCPP and SCPP_opt_ to be visualized in real time in a clinically meaningful way on the ICU monitors (Fig. 1e) [23]. Future studies are required to allow computation of SCPP_opt_ using a time window shorter than 4 h so that the injured cord is not exposed to a long course of ischemia before treatment is initiated.

The main drawback of a continuous SCPP_opt_ is that the SCPP has to vary widely within each 4-h window to define both arms of the U-shaped sPRx *versus* SCPP curve. The concept of a real-time SCPP_opt_ range for TSCI is analogous to the concept of a real-time CPP_opt_ for TBI [24, 25]. There is now strong evidence that CPP_opt_ correlates with outcome. One study [25] divided patients with acute TBI into 3 groups: those managed with CPP close to CPP_opt_ (group 1), those managed with CPP lower than CPP_opt_ (group 2, ischemia), and those managed with CPP greater than CPP_opt_ (group 3, hyperemia). Compared with group 1 patients, those in group 2 had higher mortality and those in group 3 had higher disability. Further work from large numbers of patients is needed to validate the concepts of ISP, SCPP, sPRx, continuous SCPP_opt_, etc., and to determine whether upward and downward deviations of SCPP from SCPP_opt_ are associated with worse neurological outcome.

### Complexity of ISP Signal

For a simple introduction to the concepts described in this section including complexity, edge-of-chaos dynamics, detrended fluctuation analysis, and multiscale entropy (MSE), the reader is referred to the presentation in the supplement that accompanies the article by Chen et al. [26]. The ISP signal is complex because it is influenced by many local and systemic factors that interact over different timescales, e.g., spinal cord blood flow (SCBF), tissue oxygen, tissue metabolism, and cardiac and respiratory pulsations. Complexity is a fundamental property of healthy biological systems that renders them resistant to external stress [27, 28]. Complex biological signals are characterized by self-affinity and “edge-of-chaos” dynamics. Edge of chaos means that systems transition between order and disorder, which, unlike periodicity or randomness, facilitates self-organization, evolution, and adaptability [29–33]. After TSCI, factors influencing the ISP signal become disrupted with more severe TSCIs causing more severe disruption; therefore, TSCI may be viewed as a loss of ISP signal complexity.

The nonlinear ISP dynamics can be quantified by computing hourly the detrended fluctuation exponent *α* [29, 30], the MSE [31, 32], and the maximal Lyapunov exponent *λ*_max_ [33]. Such analyses revealed that pathological processes at the injury site including cord swelling (high ISP), hypoperfusion (low SCPP), or impaired pressure reactivity (high sPRx) were associated with increased *α* and decreased MSE, which render the cord less adaptable to external changes [26]. Increased *α* indicates disrupted fractality, and decreased MSE indicates decomplexification of the ISP signal. We found negative correlations between the % of hours with edge-of-chaos dynamics (− 0.01 ≤ *λ* ≤ 0.01) *versus* high ISP and *versus* low SCPP [26]. This means that secondary insults render the ISP more regular or chaotic [26]. In a multivariate logistic regression model, better neurological status on admission, higher ISP MSE, and more frequent edge-of-chaos ISP dynamics predicted long-term functional improvement. To further access the hidden information within the complex fluctuations of the ISP signal, we mapped each ISP time series into a visibility graph [34] and quantified the topology of these graphs using concepts from complex network theory such as diameter, modularity, eccentricity, and small worldness [35]. Our data show that the topological structure of ISP graphs is highly sensitive to adverse events at the injury site, e.g., cord compression (increased ISP), hypoperfusion (reduced SCPP), and impaired vascular pressure reactivity (increased sPRx). These findings suggest that ISP signals contain clinically important information hidden within the complex signal fluctuations, not accessible with conventional signal analysis.

### ISP *Versus* Lumbar Cerebrospinal Fluid Pressure

Early attempts to obtain real-time information from the injured cord to guide management involved monitoring cerebrospinal fluid pressure (CSFP) by inserting a lumbar catheter [36] rather than monitoring ISP from the injury site [18]. Unlike pressure probes, which require surgery to place them at the injury site, lumbar catheters are widely used and easily introduced in the ICU or on the wards.

**Fig. 2:**
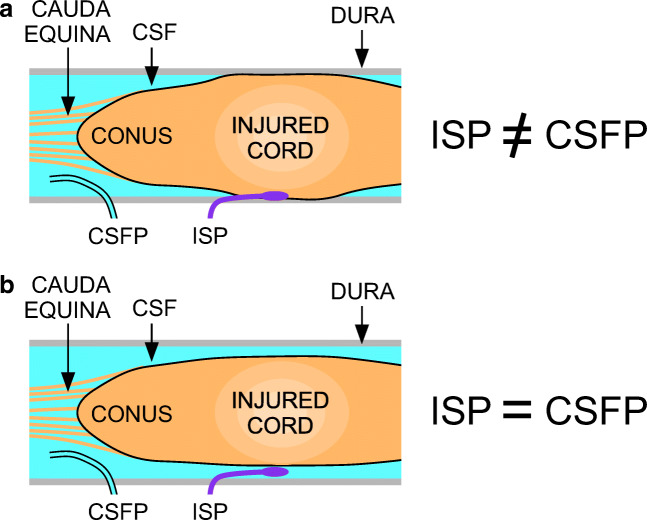
ISP *versus* lumbar CSFP. (**a**) ISP ≠ CSFP when the injured cord is swollen and compressed against the dura. (**b**) ISP ≈ CSFP when there is CSF around the injured cord.

To find out whether lumbar CSFP is the same as ISP, we simultaneously monitored CSFP and ISP in 13 patients with severe TSCI and concluded that the 2 techniques yield markedly differ values for cord pressure (ISP ≠ CSFP), perfusion pressure (SCPP_ISP_ ≠ SCPP_CSF_), and pressure reactivity (sPRx_ISP_ ≠ sPRx_CSF_) [37]. CSFP was nonpulsatile 21% of the time or had simple waveforms, whereas ISP was always pulsatile with waveforms that had the characteristic P1-P2-P3 peaks. The running correlation coefficient between ISP and CSFP was > 0.7 for > 75% of the time in 23% of patients, 25–75% of the time in 23% of patients, and < 25% of the time in 54% of patients. The extent of cord edema on MRI inversely correlated with the ISP *versus* CSFP correlation coefficient. Together, these observations suggest that cord compression against the surrounding dura may be dynamic: During periods when there is CSF around the injured cord, which indicates no compression, ISP ≈ CSFP, but when the injured cord becomes compressed against the dura, ISP ≠ CSFP (Fig. 2). The idea that cord compression against the dura at the injury site renders ISP ≠ CSFP is also supported by waveform analysis, which revealed significantly steeper δP/δT slope for ISP than CSFP and delay, by > 100 ms in most cases, between the onset of the CSFP pulse and that of the corresponding ISP pulse. These findings suggest that ISP is measured in a solid compartment (injured cord compressed against dura), whereas CSFP is in a liquid compartment. The conclusion from these studies is that ISP monitoring more accurately represents the injury site than lumbar CSFP monitoring. Lumbar CSFP monitoring may still be clinically helpful because the SCPP_CSFP_, computed as MAP-CSFP, better correlates with outcome after TSCI than the MAP [38].

## Therapies Based on ISP Monitoring

### Reducing ISP and Increasing SCPP

Several maneuvers to reduce ISP or increase SCPP have been investigated (Table 2) [18]. Reducing arterial pCO_2_ and altering the dose of the anesthetic sevoflurane or i.v. mannitol administration did not significantly affect ISP or SCPP. Increasing the dose of epinephrine increased MAP and consequently increased SCPP. Drainage of 10 mL CSF via a lumbar catheter did not significantly alter ISP in 58% of patients, significantly reduced ISP by < 5 mmHg in 33% of patients, and only significantly reduced ISP by 9 mmHg in 9% of patients [37]. Therefore, the only nonsurgical technique to reliably increase SCPP is to increase the MAP with vasopressors. The role of surgery (expansion duroplasty) in reducing ISP and increasing SCPP is discussed below.

**Table 2:** Effect of different maneuvers in TBI *versus* TSCI

Maneuver	TBI	TSCI	TSCI Ref.
Reducing arterial pCO_2_ (hypercapnia to normocapnea)	Reduces ICP, increases CPP	No effect on ISP or SCPP	[18]
Reducing sevoflurane dose	Reduces ICP	No effect on ISP or SCPP	[18]
Mannitol	Reduces ICP, increases CPP	No effect on ISP or SCPP	[18]
Hypertonic saline	Reduces ICP, increases CPP	Not tested	N/A
Vasopressors (epinephrine)	Increases MAP, thus increasing CPP	Increases MAP, thus increasing SCPP	[18]
CSF drainage	Reduces ISP, increases CPP	Little or no effect on ISP or SCPP in severe TSCI with cord compressed against dura	[37]
Surgical decompression	Reduces ISP, increases CPP, reduces mortality	Bony decompression controversial, bony + dural decompression (expansion duroplasty reduces ISP and increases SCPP)	[39, 40]

CPP = cerebral perfusion pressure; CSF = cerebrospinal fluid; ICP = intracranial pressure; ISP = intraspinal pressure; MAP = mean arterial pressure; N/A = not applicable; Ref. = references; SCPP = spinal cord perfusion pressure; TBI = traumatic brain injury; TSCI = traumatic spinal cord injury

**Fig. 3:**
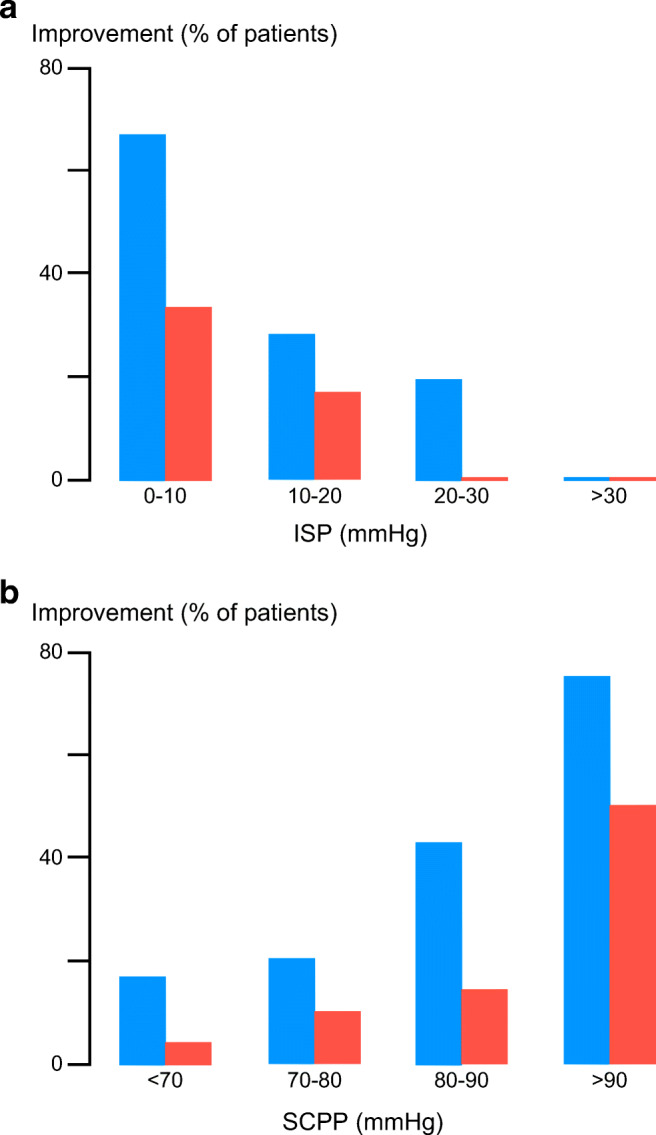
ISP and SCPP *versus* outcome. (**a**) Mean ISP and (**b**) mean SCPP *versus* % of patients that improved by at least 1 (blue) or at least 2 (red) AIS grades. Follow-up for 9–12 months.

Intervening to increase SCPP appears beneficial because it increases the amplitude of motor-evoked potentials [18] or somatosensory-evoked potentials [41] at or across the level of injury in most patients, lowers the sensory level in some patients [42], and improves limb motor responses in some American Spinal Injury Association Impairment Scale (AIS) grade C patients [18]. The chance of AIS grade conversion at 9–12 months after the TSCI negatively correlates with the mean ISP on admission and positively correlates with the mean SCPP on admission (Fig. 3) [43]. Increasing SCPP presumably reduces ischemia at the injury site. Though it is unclear whether overincreasing SCPP, such that SCPP > SCPP_opt_, worsens neurological outcome, these findings suggest that ISP and SCPP are key physiological parameters that are strongly linked to neurological status after TSCI.

### Expansion Duroplasty

**Fig. 4:**
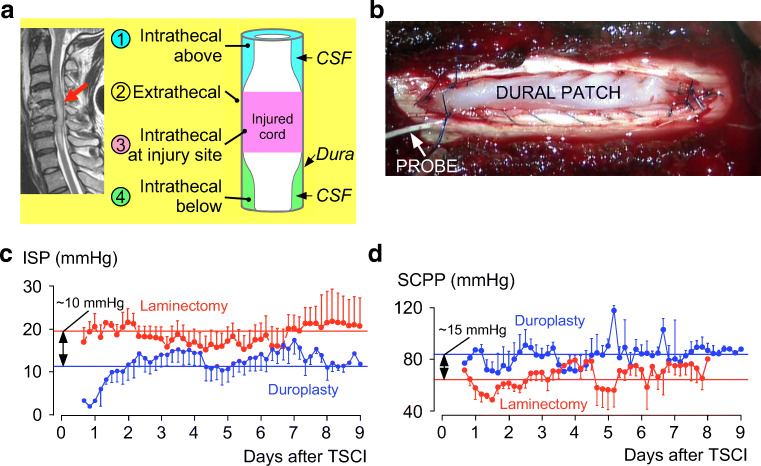
Dural cord compression and duroplasty. (**a**) MRI (*left*) and schematic (*right*). The swollen cord is compressed against the dura causing 4 compartments: intrathecal above (blue), extrathecal (yellow), intrathecal at injury site (purple), and intrathecal below (green). (**b**) Intraoperative photo of expansion duroplasty. (**c**) ISP (mean ± S.D.) and (**d**) SCPP (mean ± S.D.) *versus* days after injury for 11 patients who had bony decompression + stabilization and 10 patients who had bony + dural decompression + stabilization.

A consistent finding from ISP monitoring is compartmentalization at the injury site (Fig. 4a), which occurs because the swollen cord becomes compressed against the dura, even after adequate bony decompression [44, 45]. This is evident when monitoring pressure from several sites simultaneously, e.g., injury site (ISP), CSF compartment below, and extradural compartment: Each compartment has a different pressure [18, 46]. In an AIS grade A thoracic TSCI patient, we advanced the pressure probe intradurally, from distal to the injury site to proximal, thus defining a pressure profile with maximal pressure at the injury site [44]. Dural cord compression is also evident on MRI; in TSCI patients who had serial scans, the extend of dural cord compression resolves slowly with *t*_1/2_ ≈ 9 days [47]. These observations suggest that the dura may play a major, but unappreciated, role in spinal cord compression after TSCI.

The idea that the dura causes CNS compression is established in TBI; thus, decompressive craniectomy involves removing skull and opening dura to allow outward herniation of brain to reduce ICP and increase CPP [16, 39]. In a randomized controlled trial, decompressive craniectomy significantly reduced mortality though most surviving patients had severe disabilities [16]. In the context of TSCI, *decompression* has a different meaning from the term “decompression” for TBI. In TSCI, decompression refers to restoring normal spinal alignment and removing bony fragments or hematoma compressing the theca, i.e., extradural decompression that fails to appreciate that the cord is swollen against dura. Thus, to effectively decompress the injured cord, expansion duroplasty may be required in addition to bony decompression (Fig. 4b–d). In a pilot study, expansion duroplasty took 10–15 min to perform and was safe [48]. Compared with laminectomy in 11 TSCI patients, laminectomy + duroplasty in 10 TSCI patients reduced ISP by ~10 mmHg and increased SCPP by ~15 mmHg, on average. In the duroplasty group, 10% of patients had CSF leak, easily eliminated by placing extra sutures in ICU, and 50% of patients had noncompressive pseudomeningocele that disappeared by 6 months on MRI. Based on these findings, we plan a randomized controlled trial of expansion duroplasty for acute, severe TSCI.

### Enhancing Drug Delivery

Several trials of neuroprotective agents for TSCI have failed, despite evidence from different TSCI animal models that the drugs are neuroprotective [49–51]. In 3 TSCI patients, we injected intravenously a 4-mg bolus of dexamethasone and found that little (~0.64%, based on area under the curve calculations) dexamethasone entered the injury site [52]. The penetration of intravenously administered dexamethasone into the injured spinal cord was increased 3-fold by increasing the SCPP by 10 mmHg. This finding may explain why neuroprotective drug trials have failed: In TSCI patients,- the drug is often given in suboptimal conditions, e.g., during hypotension, which is frequently associated with cervical spinal cord injuries, thus limiting drug penetration into the injured cord. Optimization of SCPP is a prerequisite for maximizing drug delivery at the site of injury.

### Managing Fever

Fever is observed in up to 67% of patients with acute TSCI and may arise from infection or be neurogenic [53–56]. In TSCI patients, fever was associated with significantly more deranged metabolite levels than normothermia evidenced by lower tissue glucose, higher lactate, higher glutamate, and higher lactate-to-pyruvate ratio (LPR, a measure of anaerobic metabolism) [57]. Fever was particularly detrimental on injury site metabolism when the peripheral white cell count was high, which suggests that fever associated with infection may be more detrimental than neurogenic fever. In 2 TSCI patient cohorts, managed in London and Berlin [57], high fever burden correlated with less neurological improvement. Though further studies are required to determine the effect and temporal relations between the different types of fever (infection, neurogenic) and injury site metabolism, based on the data to date, we suggest prompt treatment of fever in TSCI patients with paracetamol, nonsteroidal anti-inflammatory drugs, or active cooling.

### Hypothermia

Hypothermia is being investigated as a potential therapy for TSCI [58] based on data that hypothermia is neuroprotective in animal models by targeting many pathological processes, e.g., reducing metabolic rate, inflammation, edema, oxidative stress, excitotoxicity, electrolyte imbalance, as well as apoptotic and necrotic cell death in damaged CNS tissue [59–66]. Despite the encouraging findings of animal studies, randomized controlled human trials have failed to show functional benefit of hypothermia in human TBI [67–69]. Though small, nonrandomized studies of TSCI patients suggest improved outcome after local [70, 71] or systemic [58, 72] hypothermia, there are no published randomized controlled trials of hypothermia for TSCI. A major problem with hypothermia is the paucity of mechanistic data from humans regarding the effect of cooling and rewarming on cord swelling, metabolism, and inflammation. It is thus unclear if hypothermia and rewarming have beneficial or adverse effects on the injured human spinal cord. In a study of 5 TSCI patients, a local cord hypothermia-rewarming protocol was applied. Cooling did not affect cord physiology (no change in ISP or SCPP) but markedly altered cord metabolism (increased glucose, lactate, LPR, and glutamate and decreased glycerol) and markedly reduced cord inflammation (reduced IL-1β, IL-8, monocyte chemoattractant protein (MCP), macrophage inflammatory protein (MIP)-1α, MIP-1β). Rewarming significantly worsened cord physiology (increased ICP, decreased SCPP), cord metabolism (increased lactate and LPR, decreased glucose and glycerol), and cord inflammation (increased IL-1β, IL-8, IL-4, IL-10, MCP, MIP-1α). Based on these findings, we suggest that spinal cord monitoring be employed in hypothermia studies to provide real-time information about the impact of temperature changes on spinal cord physiology and metabolism.

### Nursing Care

Nursing care after TSCI involves avoiding decubitus ulcers by frequent patient turning. Our work has shown that, after a laminectomy, external forces applied to wound are transmitted to the swollen, injured cord, causing an increase in ISP and a decrease in SCPP, thus potentially inflicting cord damage [18]. This arises because of the lack of CSF around the cord that would normally buffer the compression forces and may be important in supine patients with mid-thoracic TSCI that have a pillow placed between their shoulders [19]. Ways to prevent damaging the injured cord from external forces include avoiding wound compression and placing cross-links between the rods used to stabilize the spine.

## Multimodality Monitoring

Multimodality monitoring for TBI is based on the idea that secondary damage arises not only from altered perfusion but also from other factors such as acidosis, excitotoxicity, tissue hypoxia, and aberrant electrical activity. Thus, in TBI patients, some ICUs monitor not only ICP and CPP but also tissue metabolism (hourly tissue glucose, lactate, pyruvate, LPR, glutamate, glycerol) using MD [73], tissue oxygen using a Licox probe [74], hemoglobin saturation using near-infrared spectroscopy [75], and spreading depolarizations using electrode arrays [76].

### Microdialysis

**Fig. 5:**
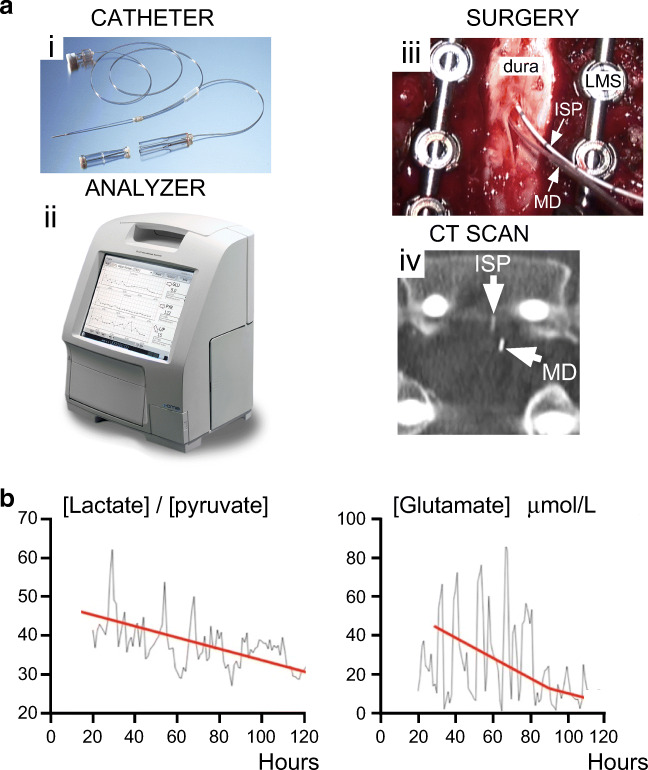
Multimodality monitoring after TSCI. (**a**) Setup for ISP + microdialysis monitoring: i) microdialysis catheter, ii) microdialysis analyzer, iii) intraoperative photo showing ISP probe + microdialysis catheter, and iv) postoperative CT showing ISP probe + microdialysis catheter. (**b**) LPR (*left*) and glutamate (*right*) *versus* time for a TSCI patient.

In TBI, there is substantial evidence that derangement of tissue metabolism correlates with outcome [73]. We have recently reported a technique to monitor spinal cord metabolism after TSCI with surface MD (Fig. 5) [52]. Studies in pigs show that surface and intraparenchymal MD give comparable metabolite values in the pulsating heart [77] and liver [78]. For spinal cord, surface MD at the injury site differs markedly from corresponding measurements taken from the lumbar CSF [37]. The key findings of our study are that SCPP strongly correlates with injury site metabolic profile and that the extent of metabolic derangement and the probability of AIS grade conversion after TSCI correlate well with the degree of metabolic derangement at the injury site.

A problem with multimodality monitoring is interpretation of the large volumes of data in a clinically meaningful way. In TSCI patients, we analyzed the MD data using Kohonen self-organizing maps and discovered 3 metabolic patterns termed near-normal, ischemia/necrosis, hyperemia, and distal [79]. “Big data analytics,” currently under development to extract conclusions from large datasets [80], could be applied to evaluate multimodality data. Additional techniques, e.g., Granger [81] or Sugihara [82] causality analysis, which quantify directions of information flow in time series may also be useful. Suppose there is an increase in LPR, fall in tissue oxygen, and rise in ISP. Causality analysis may be used to determine whether the rise in ISP caused the fall in tissue oxygen, which, in turn, caused the rise in LPR. Knowing the direction of information flow is clinically useful to differentiate between cause and effect. Further studies are awaited in TSCI patients to determine the clinical value of additional monitoring from the injury site including tissue oxygen and spontaneous electrical activity.

### Spinal Cord Blood Flow

**Fig. 6:**
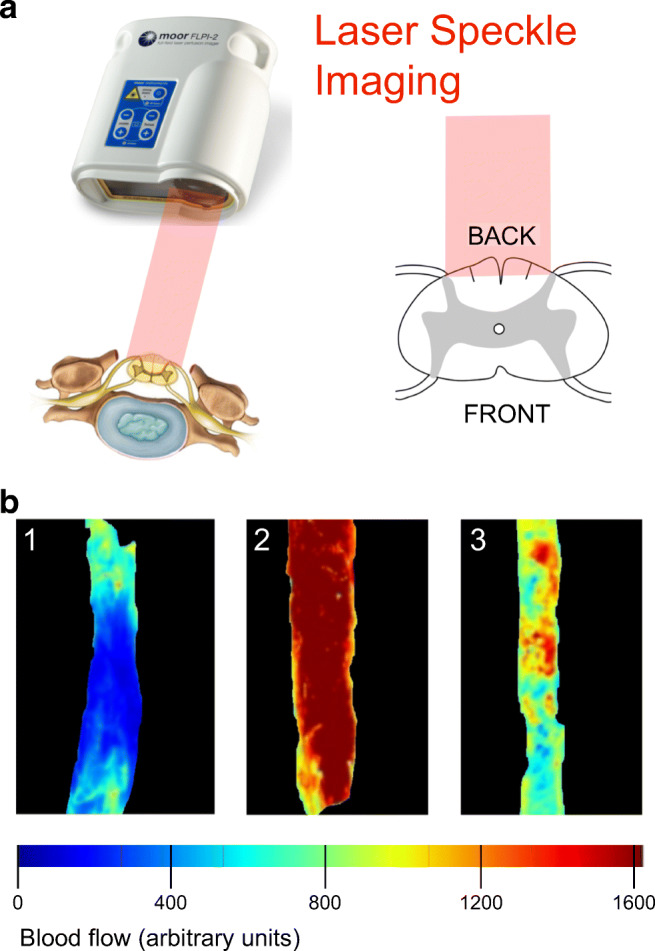
Imaging spinal cord blood flow during surgery. (**a**) Schematic of setup. Laser speckle imager, infrared laser (red beam), and dorsal theca exposed after laminectomy. (**b**) Three patterns of spinal cord blood flow after injury (*upper*) termed as 1) necrosis-penumbra, 2) hyperperfusion, and 3) patchy perfusion. Spinal cord blood flow scale (*bottom*).

The impact of TSCI on SCBF in humans is poorly understood. Advanced MRI techniques are limited largely due to artifacts from cardiorespiratory motion as well as signal loss from the bone and the metalwork used to stabilize the spine. In a recent study, we investigated SCBF intraoperatively using laser speckle contrast imaging, a noninvasive technique in which a laser beam penetrates through the dorsal theca, 2–3 mm deep into the spinal cord, thus imaging blood flow in the dorsal columns [22]. We discovered 3 SCBF patterns, characterized by distinct injury site metabolic signatures: necrosis-penumbra, hyperperfusion, and patchy perfusion (Fig. 6). In some TSCI patients, increasing the MAP by 20 mmHg increased the overall SCBF at the injury site, though blood flow increased in some regions but decreased in others. This phenomenon, termed blood pressure–induced local steal, may partly explain the detrimental effect of hyperperfusion. Further studies are required of real-time SCBF monitoring in the ICU as part of multimodality monitoring.

## Future Directions

Monitoring spinal cord physiological and biochemical parameters from the injury site allows individualized, targeted perfusion therapy in TSCI patients, e.g., eliminating hypoperfusion or optimizing drug delivery. To date, monitoring data after TSCI have only been obtained in 1 center only (St. George’s in London); it is important for other centers to independently validate these findings. An important finding is that the dura causes cord compression and thus a randomized controlled trial of expansion duroplasty for TSCI is being set up.

The probes used in our studies were designed for TBI; future designs may allow the probes to be inserted in TSCI patients in ICU without surgery. The ideal TSCI pressure probe should be radio-opaque and MRI compatible and have several measuring points to define the pressure profile of the injured cord. Such a probe will provide several ISP, sPRx, and SCPP readings simultaneously, which would require the concept of SCPP_opt_ to be redefined. An alternative to inserting probes intradurally is noninvasive, transcutaneous monitoring of hemoglobin oxygenation by near-infrared spectroscopy [40], although beam scatter by skin, muscle, bone, and metalwork may hinder such techniques. The recent availability of online MD, which allows continuous monitoring of extracellular tissue glucose, lactate, and pyruvate, may reveal novel pathological phenomena that occur at the timescale of seconds rather than hours. Finally, the monitoring techniques described here may also be applied in conditions associated with cord edema other than TSCI, e.g., neuromyelitis optica [83].

## Electronic supplementary material

ESM 1 (PDF 1224 kb)
